# Comparison of different human tissue processing methods for maximization of bacterial recovery

**DOI:** 10.1007/s10096-018-3406-4

**Published:** 2018-10-24

**Authors:** Mohamed Askar, Waheed Ashraf, Brigitte Scammell, Roger Bayston

**Affiliations:** Department of Academic Orthopaedics, Queen’s Medical Centre, University of Nottingham, C Floor, West Block, Derby Road, Nottingham, NG7 2UH UK

**Keywords:** Homogenization, Tissue processing

## Abstract

Tissues are valuable microbiological samples that have proved superiority over swabs. Culture of tissue samples is used in the diagnosis of a variety of infections. However, as well as factors such as the site of obtaining the sample, the number of samples, and previous antibiotic use, the method of tissue processing may have an important effect on sensitivity. Data from the literature comparing different tissue processing methods is very limited. This study aimed to compare different mechanical and chemical methods of tissue processing in terms of efficacy and retaining the viability of the bacteria in the tissues. Standard suspensions of *Staphylococcus aureus* and *Escherichia coli* were prepared and treated differently to test the effect of that treatment on bacterial viability. Artificially inoculated pork tissue and known infected human tissue samples were then processed by different methods prior to culture, and results were compared. Percentages of reduction in the number of viable bacteria compared to the control by homogenization was similar to 5-min dithiothreitol treatment but significantly lower than bead beating. Bacterial recovery from homogenized human tissues was significantly higher than from any other method of treatment. Although bead beating could be the most efficient method in obtaining a homogeneous tissue product, it significantly reduces the number of viable bacteria within tissues. Homogenization offers the most effective easily controllable retrieval of bacteria from tissue and retains their viability. Guidelines for diagnosing infections using tissue samples should include a standardized processing method.

## Introduction

Tissue samples are among the most valuable samples for microbiological testing, particularly in musculoskeletal infections. Recovery of bacteria from tissues has been included in many guidelines for diagnosis of different infectious diseases. An example is the Musculoskeletal Infection Society (MSIS) definition of prosthetic joint infection (PJI) [1]. Furthermore, tissue samples have a proven superiority over swabs as a diagnostic material [2]. Several factors can affect the quality and reproducibility when using tissues for diagnosing infections, e.g., method of sample collection and number of samples [3]. Previous antibiotic use and the method of tissue processing could also have a detrimental effect on sensitivity [4]. The quality of every subsequent step in diagnosing infections is dependent on the effectiveness of the initial step of tissue processing. For highly sensitive microbiological analyses, it is crucial to ensure maximal bacterial release from tissues.

There are currently several tissue processing methods ranging from simple direct streaking on agar plates or manual partitioning with scalpels to more automated homogenizers and chemical lysis, yet there is no firm consensus regarding the efficacy of such processing methods in terms of tissue disruption, releasing of bacteria from tissues, and the compromise of bacterial survival with each method.

In this study, different mechanical and chemical processing methods were compared in vitro and ex vivo to determine their efficacy and reproducibility.

## Methods

### Exposure of bacterial suspensions to processing

In order to determine the impact of three different methods on viability of planktonic bacteria, suspensions were subjected to homogenization or bead beating for various times.

*Staphylococcus aureus* and *Escherichia coli* previously isolated in the Biomaterials-Related Infection laboratory, University of Nottingham, UK, were used for the preparation of bacterial suspensions in tryptic soy broth (TSB, Oxoid, Basingstoke, UK) at a concentration of 10^5^ cfu/mL. Concentration was controlled by light absorbance calibration and plating of serial dilutions.

One hundred microliters of each suspension was added to 1 mL of phosphate-buffered saline (PBS) and treated by either homogenization for two or four cycles (each cycle lasting for 45 s at a speed of 4500 rpm with 45 s between cycles) or bead beating for two or four cycles in the same way as homogenization using 0.1-mm glass beads. The MagNA Lyser homogenizer (Roche, Basel, Switzerland) was used.

One hundred microliters of bacterial suspensions were also added to 0.1% *w*/*v* dithiothreitol (DTT, Thermo Fisher Scientific, Vilnius, Lithuania) in sterile water for 5 min before plating to be compared to homogenized samples for four cycles as described above.

One hundred microliters of each of processed and control (unprocessed) bacterial suspension was spread on blood agar plates (Oxoid), and colonies were counted after overnight incubation.

### Mechanical processing of artificially inoculated pork meat

To determine the influence of tissue on bacterial viability during processing, pork tissue samples were “spiked” with bacterial suspensions and processed by three methods. Pork boneless meat was obtained from a local grocery store. Equal pieces weighing 7.5 g were prepared. Each piece was thoroughly and repeatedly washed in sterile water and further divided into equal thirds (2.5 g each), and stored in a − 20 °C freezer for future use.

One hundred microliters of 10^5^ cfu/mL bacterial suspension (*S. aureus* or *E.coli*) was used for inoculation at the center of the meat piece using a 1-mL plastic syringe (Becton Dickinson, Drogheda, Ireland) and 16-mm, 5/8″ needles (Becton Dickinson).

A third meat piece was left “blank” without inoculation to report on possibility of contamination.

After inoculation, samples were left on the bench for 2 h then processed by either homogenization, bead beating, or vortexing. Aliquots of 0.5 g of each piece were transferred to 2-mL tubes along with 1 mL of PBS. For bead beating, tubes contained 0.1-mm glass beads (Cambio: UC-13118-50; Glass Beads; 0.1 mm). Four cycles of homogenization and bead beating were run, 45 s each at 4500 rpm with 45 s in between. Vortexing was used to simulate the routine tissue processing in many microbiology laboratories. This was the same as the tissue homogenization tube but used for vortexing for 20 s instead of homogenization.

One hundred microliters of each product was plated out and incubated overnight. Colonies were counted and recorded. For the results to be considered valid, the blank piece plates had to grow neither *S.aureus* nor *E.coli*, though they may have other irrelevant bacteria despite the prior extensive rinse.

### Known infected human tissues

To compare the various processing methods when applied to infected human clinical material, 26 tissue samples were collected from six patients diagnosed with PJI according to the MSIS criteria. 0.5 g of each sample was added to 1 mL of PBS and processed by either homogenization, bead beating, vortexing, sonication, DTT, or proteinase K treatment. Homogenization, bead beating, vortexing, and DTT were run as described above. For proteinase K (Roche, Mannheim, Germany), 20 μL were added 10 min before plating. One tube was sonicated for 5 min at 50 Hz. After it became clear that homogenization and DTT processing were superior, 14 known infected human tissue samples were collected from either diabetic feet [4] or prosthetic joint infections with pus-draining sinuses [5] and were processed by either homogenization or DTT only.

The liquid product of each processing was cultured on blood agar plates and incubated aerobically and anaerobically for 72 h and 14 days respectively.

### Statistical analysis

Graphpad Prism 7 was used to analyze data and produce charts. Appropriate statistical tests were used according to the distribution of data. *P* values < 0.05 were considered significant.

### Ethics

Human tissue samples were collected under the ethical approval of the Nottingham Health Science Biobank.

## Results

### Bacterial suspensions exposed to processing

**Fig. 1:**
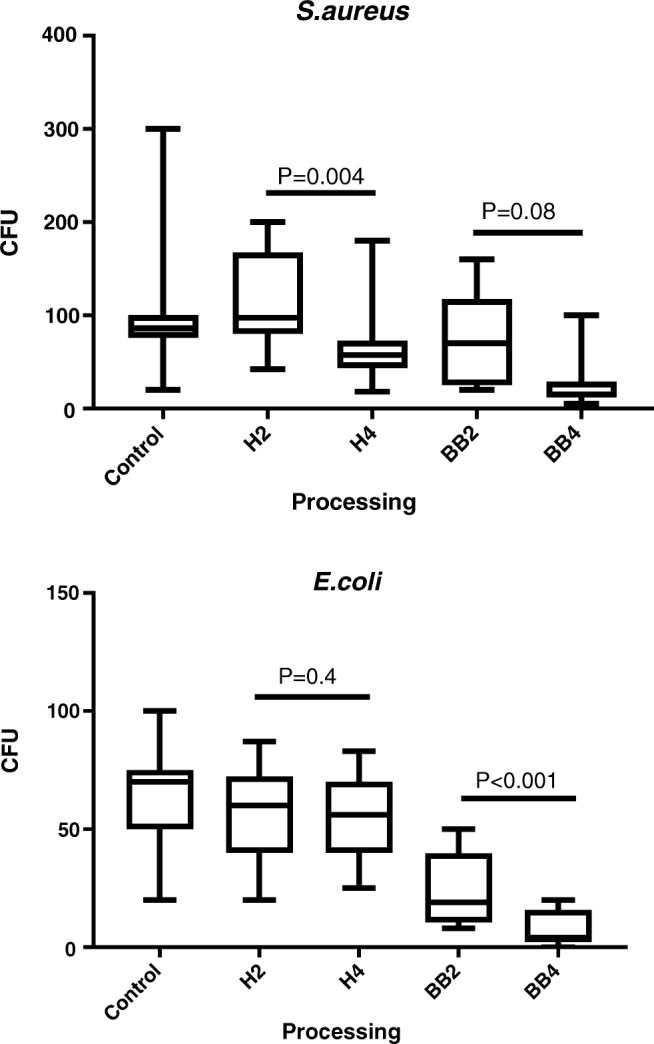
Bacterial recovery from *S.aureus* and *E.coli* suspension after homogenization and bead beating. H2 and H4 are two and four cycles of homogenization respectively; BB2 and BB4 are two and four cycles of bead beating respectively. CFU colony forming unit

**Fig. 2:**
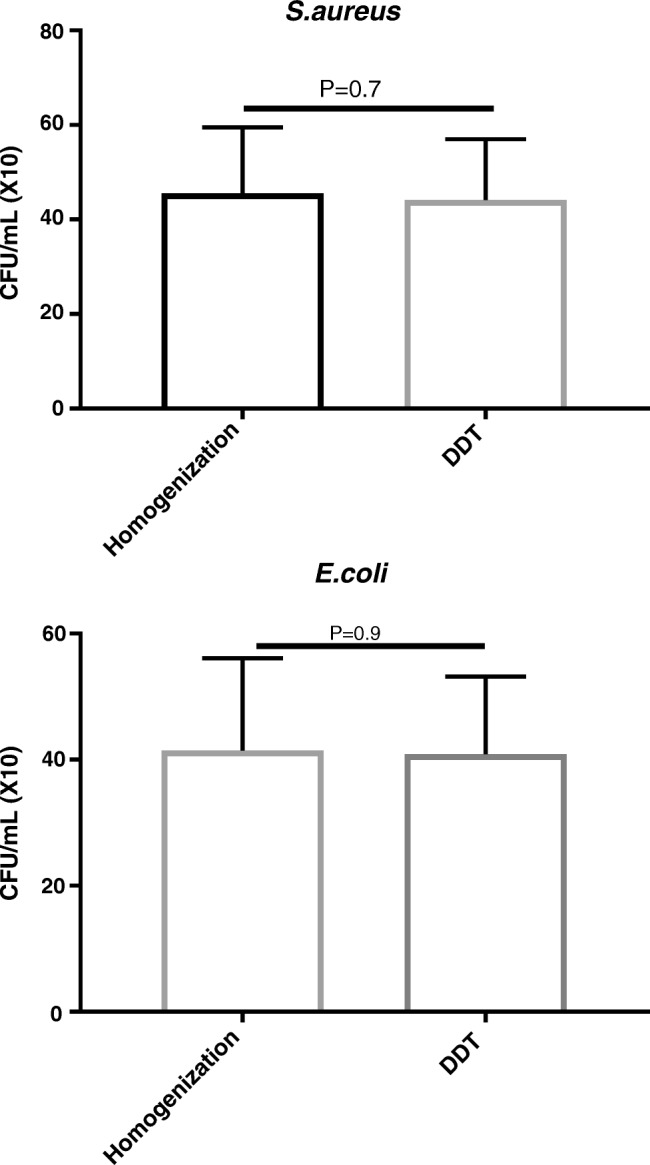
Bacterial recovery from *S.aureus* and *E.coli* suspension after homogenization versus DTT

Quantitative bacterial recovery from any processed bacterial suspension was significantly lower than from the unprocessed control group. The percent reductions compared to unprocessed suspensions are shown in Table 1. Bacterial recovery from bead-beaten samples was lower than from homogenized samples. The difference in reduction between two and four cycles of homogenization was statistically significant for *S.aureus* but not for *E.coli* (Fig. 1). There was no statistically significant difference between bacterial suspension samples treated by either homogenization or DTT as shown in Fig. 2.

**Table 1:** Percentages of reduction in bacterial count after different processing

Organism	Processing variable	Homogenization		Bead beating	
		2 cycles	4 cycles	2 cycles	4 cycles
*S.aureus*	Median	14	35	48	80
	interquartile range	2–24	24–43	33–60	70–84
*E.coli*	Median	10	13	67	92
	interquartile range	0.5–20	5–20	54–76	83–96

### Mechanical processing of artificially inoculated pork meat

**Fig. 3:**
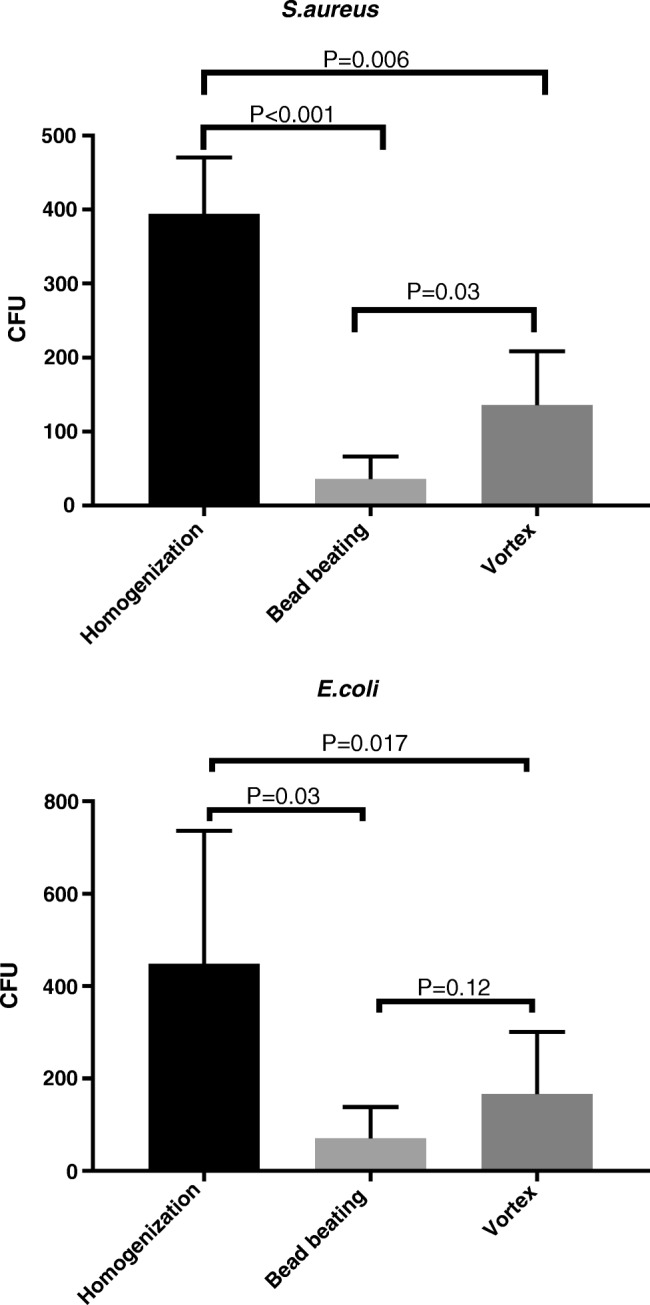
Quantitative recovery of *S.aureus* and *E.coli* from inoculated pork samples by homogenization, bead beating, and vortexing. CFU colony forming unit

Quantitative bacterial recovery from artificially inoculated pork samples differed among samples depending on whether they were homogenized, bead beaten, or vortexed. For *S.aureus*, means of recovered bacteria by homogenization, bead beating, and vortexing were 394, 36, and 136 cfu/mL respectively. For *E.coli*, means were 448, 70, and 166 cfu/mL respectively. Results are shown in Fig. 3.

### Known infected human tissues

**Fig. 4:**
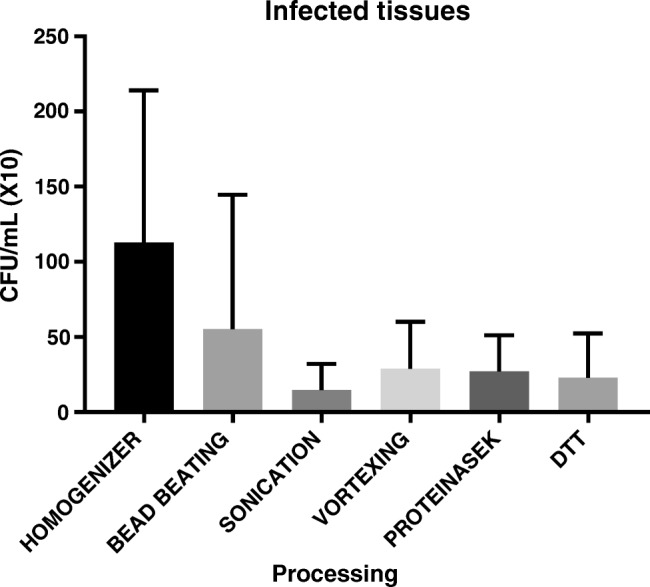
Quantitative recovery from human infected tissue samples (from PJI cases) processed by different methods. Bacterial recovery was significantly higher with homogenization (*p* = 0.024)

**Fig. 5:**
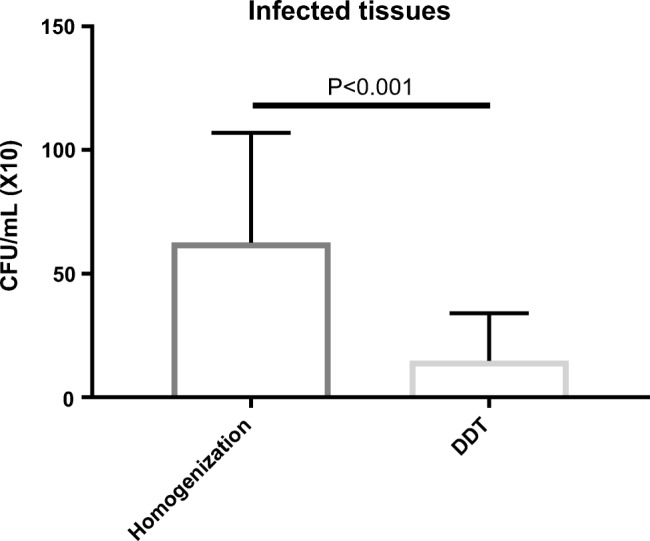
Quantitative recovery from infected human tissue samples (from four diabetic feet and ten PJI cases) processed by homogenization versus DTT

Nine out of the 26 tissue samples were positive by at least one processing method. Only one patient with PJI had negative culture results with all processing methods. Quantitative bacterial recovery from infected human tissues were significantly higher (*p* = 0.0239) after homogenization than all other samples (Table 2 and Fig. 4). Bacterial recovery from human-infected tissues from cases of diabetic feet and PJI was significantly higher with homogenization than DTT (Fig. 5). Table 3 summarizes the results of homogenization versus DTT.

**Table 2:** Bacterial recovery in cfu/100 μL from infected human tissue samples processed by different methods (*H*, homogenization; *BB*, bead beating; *S*, sonication; *V*, vortexing; *PK*, proteinase K; *DTT*, dithiothreitol)

Sample	Organism	H	BB	S	V	PK	DTT
1	*Staphylococcus epidermidis*	5	0	0	0	1	0
2	*Enterococcus faecalis*	180	68	15	92	40	91
3	*Enterococcus faecalis*	139	68	19	21	44	22
4	*Enterococcus faecalis*	174	60	48	32	32	36
5	*Enterococcus faecalis*	300	280	16	59	71	26
6	*Staphylococcus epidermidis*	4	0	0	1	1	0
7	*Enterococcus faecalis*	5	4	0	0	3	0
8	*Enterococcus faecalis*	66	10	35	41	38	31
9	*Staphylococcus epidermidis*	142	8	0	13	14	2

**Table 3:** Bacterial recovery from infected tissue samples processed by homogenization versus DTT (cfu/100 μL)

Sample	Infection	Organism	Homogenization	DTT
1	Diabetic feet	*S.epidermidis* *Corynebacterium pseudotuberculosis*	53	4
2	Diabetic feet	*S.aureus*	45	2
3	Diabetic feet	*Streptococcus dysgalactiae*	128	12
4	Diabetic feet	*Serratia marcescens* *Staphylococcus capitis*	140	13
5	PJI	*Staphylococcus epidermidis*	3	1
6	PJI	*Enterococcus faecalis*	125	74
7	PJI	*Enterococcus faecalis*	56	16
8	PJI	*Enterococcus faecalis*	78	7
9	PJI	*Enterococcus faecalis*	35	32
10	PJI	*Enterococcus faecalis*	2	0
11	PJI	*Enterococcus faecalis* *S.aureus*	12	0
12	PJI	*Enterococcus faecalis* *S.aureus*	58	17
13	PJI	*Enterococcus faecalis* *S.aureus*	73	13
14	PJI	*Enterococcus faecalis* *S.aureus*	68	16

## Discussion

The first steps of tissue sample processing have a great impact on the downstream steps in the microbiological workflow. For diagnosis of infections in which recovery of bacteria from infected tissues is a gold standard, the ability to release the bacteria is crucial. As the majority of bacteria are not expected to be on the outside surfaces of collected tissue samples, it is not sufficient just to streak tissues on agar plates or to immerse them in culture broth. Microbial distribution and loads within the potentially infected tissues can vary widely. Depending on whether the dissection and harvest planes will cross a focus of bacterial collection, some microbes could be located on the surface of the tissues and might be retrieved by direct streaking or swabbing of the tissues, but it is just as likely that surface contaminants are isolated.

Prior steps of releasing bacteria should be carried out first to enhance the sensitivity of the microbiological tests. There are two prerequisites for an ideal processing method: to release as many bacteria as possible and not to adversely affect bacterial viability. Here, we demonstrate a “tissue processing paradox” in which the higher the force delivered to tissues for processing, the more homogeneous the end product, and apparently, the more bacterial release, but the lower bacterial survival. A balance should be achieved to ensure both maximal release and maximal viability of bacteria. As quantitative rather than qualitative estimation of bacteria in tissues is crucial for guiding the treatment, the method of initial tissue processing is therefore important.

Diagnosis of prosthetic joint infection (PJI), for instance, has been and is continuing to be challenging for both microbiologists and surgeons with tireless efforts to improve and standardize it. Periprosthetic tissue samples collected at the time of surgery are among the most valuable materials for PJI diagnosis [6, 7]. One of the two major criteria of the MSIS definition of PJI depends on the isolation of the pathogen from tissue or fluid samples [1]. In that consensus, different tissue-related aspects were discussed such as how many samples should be collected, how they should be collected, and for how long cultures should be incubated, but no mention is made of tissue processing methods. The tissue processing method could have a detrimental effect on the accuracy of microbiological diagnosis of PJI, but data in the literature on the optimal tissue processing method are limited.

The optimal number of samples for diagnosis of PJI has been addressed by a number of studies. Atkins et al. 1998 [3], Bemer et al. 2016 [8], and Peel et al. 2017 [9] recommended five to six, four, and three to four samples respectively. They processed tissue samples by manual bead milling, automated bead milling, and homogenization respectively. Different tissue processing could be one possible explanation for the variability of their results. Atkins et al. [3] developed a mathematical model to reach their conclusion and recommendation of five to six tissue samples to be collected with a diagnostic cutoff of three or more positive cultures for satisfactory sensitivity and specificity. Bemer et al. [8] conducted a multicenter study and recommended four samples to be collected and cultured on three different media for an accurate cost-effective diagnosis of PJI. Peel et al. [10] found that culturing of tissue samples in blood culture bottles could enhance the sensitivity of PJI diagnosis without compromising the specificity. Later, they concluded that three tissue samples cultured in blood culture bottles can give the same level of accuracy as four samples cultured conventionally [9]. It is clear that much care was given to the culture media which are unarguably crucial for enhanced bacterial recovery. Yet, initial tissue disruption has not received the same attention in spite of the potentially equal impact.

Furthermore, culture-negative PJI can occur in approximately 7% of all PJI cases, and it remains a challenge and a source of frustration for both microbiologists and orthopedic surgeons [5]. In our study, one patient had negative culture results despite the processing method. A well-studied reason for failure of culture to retrieve the causative organism is prior antibiotic use [5, 11]. However, suboptimal microbiological methods could also be implicated especially with low tissue microbial load. In our case, the patient had not been on antibiotics prior to surgery, and different processing methods did not improved the detection of causative organism which might have been in a viable but non-culturable state [12].

The ideal tissue processing method should have the highest possible bacterial release and survival. In other words, it should not be too harsh for the bacteria to survive. In addition, it should be less liable to contamination and not over-dependent on worker’s skills.

In the present study, the ability of the processing method to keep the bacteria alive and cultivable was firstly addressed by processing bacterial suspensions and comparing them to the control non-processed group. Homogenization showed the highest bacterial survival.

An example of gram-positive and gram-negative common pathogens was chosen to be tested. The effect of processing can vary among different bacterial species and strains, most probably because of variability in their cell envelope structure and hence resistance to mechanical stress. Two and four cycles of homogenization and bead beating were tested. As toughness of different tissues is variable, the amount and duration of the force that needs to be delivered to obtain a homogeneous mixture can vary. Tissue processing should always be kept to the minimum required as the longer the processing, the lower the viability of the released bacteria.

In order to determine whether this conclusion held true in case of infected tissues, artificially inoculated pork tissues were processed, and the same result was found. Pork was chosen because of presumptive similarity with human tissues [13].

DTT has been recommended for tissue processing because of its high sensitivity, specificity, practicability, and relatively low cost [14, 15]. In this study, DTT was found to be as safe as homogenization in terms of bacterial viability. However, when they were compared in terms of bacterial release from known infected tissue samples, homogenization was significantly better. Lack of this comparative study was admitted as a limitation by the authors recommending use of DTT [15]. Nevertheless, DTT could be considered as an alternative in case of unavailability of homogenization because of its preservation of bacterial viability.

As a final step, tissue samples collected from PJI patients diagnosed according to the MSIS criteria were used to compare homogenization, bead beating, sonication, vortexing, proteinase K, and DTT treatment. Homogenization showed significantly higher bacterial release. The enhanced quantitative and qualitative microbial yield using homogenization has been reported previously [4]. Manual and automated bead beating has also been described in PJI diagnosis [3, 8, 16]. In our study, bead beating, in spite of a very homogeneous product, seems to be destructive to the released bacteria with low bacterial count end result. Sonication has a good reputation in releasing bacteria from biofilms from the surfaces of the implants [17–20] but this method does not give a homogeneous product in the case of tissues.

Our study confirms the results of Redanz et al. 2015 [4] but gives a clearer view of the superiority of homogenization firstly by giving an estimate of loss of bacterial viability using different processing methods. Secondly, this is a comparative study between different published mechanical and chemical methods. The study by Redanz et al. 2015 [4] compared homogenization to direct plating which is not a suitable alternative because of lack of actual processing.

The current study has number of limitations. First, the small number of samples might make it difficult to generalize the conclusion. Second, the study did not include fungi or anaerobic bacteria which are uncommon, yet reported, causative organisms of PJI, and other infections. Lack of isolation of gram-negative bacteria from infected human tissues also might make it hard to generalize these findings. However, these results give good evidence of superiority of homogenization in releasing bacteria while retaining their viability.

In conclusion, homogenization offers the most effective retrieval of bacteria from tissue and retains their viability. Tissue processing should be standardized and included in guidelines of diagnosis and definition of PJI and other infections because of the detrimental effect of some methods.
